# Integrated Fruit Production and Pest Management in Europe: The Apple Case Study and How Far We Are From the Original Concept?

**DOI:** 10.3390/insects6030626

**Published:** 2015-06-26

**Authors:** Petros Damos, Lucía-Adriana Escudero Colomar, Claudio Ioriatti

**Affiliations:** 1 Open University of Cyprus, Faculty of Pure and Applied Sciences, Department of Environmental Conservation and Management, Main OUC building: 33, Giannou Kranidioti Ave., 2220, Latsia, Nicosia, Cyprus; 2 IRTA, Sustainable Plant Protection (Entomology), IRTA-Mas Badia Agricultural Experimental Station. La Tallada d’Empordà S/N. 17134, Girona, Spain; E-Mail: adriana.escudero@irta.cat; 3 Technology Transfer Centre, Fondazione Edmund Mach, Via Edmund Mach 1, 38010 San Michele all’Adige (TN), Italy; E-Mail: claudio.ioriatti@fmach.it

**Keywords:** Integrated Pest Management, Technical Guidelines, Sustainable Pest Management, pest control tools

## Abstract

This review focuses on the process of adapting the original concept of Integrated Pest Management (IPM) to the wider conception of the Integrated Fruit Production (IFP) implemented in Europe. Even though most of the pest management strategies still rely on the use of synthetic pesticides, a wide array of innovative and environmentally friendly tools are now available as possible alternative to the pesticides within the modern apple production system. We also highlight how recent pest management strategies and tools have created an opening for research towards IPM improvement, including the use of biorational pesticides, semiochemicals and biological control. Forecasting models, new tree training systems and innovative spray equipment have also been developed to improve treatment coverage, to mitigate pesticide drift and to reduce chemical residues on fruits. The possible threats that jeopardize the effective implementation of IPM and particularly the risks related to the development of the pesticide resistance and the introduction of new invasive pests are also reviewed. With the directive 128/09, the European legislation recognizes IPM as a strategic approach for the sustainable use of pesticides. Within this context, IPM and related guidelines is called to meet different areas of concern in relation to the worker and bystander safety. Beside the traditional economic criteria of the market-oriented agriculture, sustainable agriculture includes the assessment of the environmental impact of the agronomic practices within the societal context where they take place. As a consequence of the raising consumer concerns about environmental impacts generated by the fruit production, IFP certification over product standards, including process aspects, are frequently required by consumers and supermarket chains.

## 1. Introduction

Historically, pest control in fruit orchards was based on broad-spectrum pesticides which were associated with a diversity of problems, traditionally including environmental effects, beneficial organism extinction, and pesticide resistance. More recently, other potential negative effects caused by the use of pesticides in agriculture have drowned the attention of the public and policy makers: the threat toward the pesticide applicators and bystanders’ health and the food safety endangered by pesticide residues. Fears about these issues have increased the interest in the development of alternative means for pest control that causes trivial or no impact on humans, beneficial organisms and sensitive ecosystems [1,2]. Nevertheless, most pest management programs still rely heavily upon the role of conventional insecticides which are in most cases the principal means for pest and disease control in fruit orchards [3,4,5]. In apple production, particularly, the presence of arthropod pests, several serious diseases, and high cosmetic standards for fresh fruit market (e.g., there is zero tolerance for the live codling moth) represent formidable obstacles to the adoptions of alternative means of pest control and low-spray apple production. Nevertheless, increased public fears about the role of pesticides and the potential adverse effects on human health, wildlife, soil water, and overall environmental quality have led to the development of alternative low risk control tools [6].

The solution was found in adopting and promoting a concept already formulated in the late 50s and called “Integrated Pest Management” (IPM). The turning point with regard to the definition of the concept of IPM is considered to be 1958 [7] when two separate study groups, one American and one Dutch, looking for alternative solutions to the defense of crops introduced the concept of integrated control at the 10th International Congress of Entomology held in Montreal. The document arose from concern that arises from the obvious deleterious effects caused by broad-spectrum insecticides used in agriculture [8,9]. The term “integrated control” proposed by researchers in California [10,11] was unanimously accepted by the international community. The concept worked out by Stern *et al.* in 1959 [11] and in principle is integrating the role of biological and other control measures in complementary ways. They provided the foundations for understanding the relationship between the damage and the beneficial insects in agricultural systems and how the farmer could exploit them to their advantage through the detailed description of the equilibrium position among populations of pests, the economic threshold (ET), and the economic injury level (EIL).

In Europe, noteworthy was the role played since 1956 by the International Organization of Biological Control (IOBC), and more specifically, the pioneering activity of fruit entomologists operating within the IOBC working group “integrated control in orchard” established in 1959 with the aim of promoting the development of biological control in a comprehensive concept of integrated fruit production [12].

A few years later, in 1962, van den Bosch and Stern [13] extended the conceptual framework of IPM to embrace the coordinated utilization of all available biological, cultural, and artificial practices.

In 1965 the concept was explicitly specified during a symposium sponsored by the Food and Agriculture Organization (FAO), of the United Nations, held in Rome, Italy [14,15] and with the participation of 87 delegates from 34 countries and seven international agencies [16].

Since then, national and international institutions as well as fruit cooperatives and grower associations has been encouraging farmers to move towards IPM techniques to minimize pesticide use and trim costs, supported by the continuous improvements in monitoring systems and in the basic understanding of pest biology and pest-plant interactions [6].

The development occurring during the early 1970s brought a change in the general concept and IPM become an indispensable element of a holistic system approach involving the entire orchard and defined as Integrated Fruit Production (IFP). On this virtuous route, the message of Ovronnaz, launched in 1976, was a cornerstone of the modern IFP because it introduced the need to abandon the isolated view of plant protection (IPM) and to place it in the context of the entire farm [12]. IFP and IPM offer an economical and high quality fruit production framework, giving priority to ecologically safer methods, minimizing the undesirable side effects and use of agrochemicals, and enhancing the safeguard of the environment and human health [17,18,19,20,21].

Consumer demands for certified products have a significant impact on current fruit production. Recent market studies [22] show that the European consumers are willing to pay for the reduction of pesticides and apple benefits of a premium price when produced under IPM of organic certified process. Nevertheless, there are also cautions weather IPM programs that are sustainable in the longer term and they will continue to evolve, applying an increasingly wider range of new settings [23,24,25]. Nevertheless, despite these, IFP is recognized as a model of sustainable plant protection in all European countries [19].

Until recently, the application of IPM was a voluntary approach implemented by the most advanced and environmental friendly fruit production systems. Since the approval of the EU Directive 128/2009 that established a framework for Community action to achieve the sustainable use of pesticides, the adoption of IPM becomes compulsory in Europe. In fact, member states are required to adopt National Action Plans to set up quantitative objectives, targets, measures and timetables to reduce risks and impacts of pesticide use on human health and the environment and to encourage the development and introduction of IPM and of alternative approaches or techniques in order to reduce dependency on the use of pesticides.

Within this context, IPM has changed its original goal and it is called to cover different areas of concern including worker protection, pesticide residues on fruits and overall protection of the environment and society.

The current review describes some recent advances in Integrated Fruit Production with special reference to Pest Management in Europe. The apple production systems are employed as a case study and emphasis is given on what has been changed in the apple production system since the first definitions of IPM. Moreover, stress is given in control methods used to manage apple key pest and diseases, including: the use bio-pesticides, semiochemicals and biotechnical measures, biological control, decision tools and models. Lastly, a discussion is drawn on how these tools may be transformed to proxies based on the implementation of recent guidelines, market requirements and the skeletal system of the EU directives.

## 2. Current IPM Tools and Techniques Used in Integrated Fruit Production

IPM combines knowledge of the pest and host plant with multiple tactics for long-term pest control. It is consistent with sustainable agriculture and uses total farm systems approaches to mitigate pest pressure. Sustainable agriculture provides a conceptual framework and fundamental principles to guide the future development of husbandry practices and arrangements that are economically viable, environmentally sound, and socially responsible. In this context, various authors have advocated the principle of incorporating all available control methods and measures to manage pests that are being chosen by environmental, economic and societal standards. Currently, the term IPM incorporates the wide array of pest management practices, which are adopted following certain criteria, included into a total system approach which ideally should take on all crop production objectives [20,25,26].

### 2.1. Pesticides

Although IPM uses all available means to keep pest populations below levels that would cause economic loss, most IPM programs still rely heavily upon the use of pesticides. Yet, recently the European commission launched a course of study which has filled in the reassessment of a list of conventional pesticides that were on the markets before 1993 [27]. The evaluation program concerned about 1000 substances and has led to removal from the market of more than two thirds of these [27,28]. Particularly, 26% of the reviewed pesticides (corresponding to approximately 250 substances), have topped the EU harmonized safety assessment, while the vast majority (67%) has been eliminated because evaluation dossiers were either not submitted, incomplete or withdrawn by the chemical industry, while nearly 70 substances failed the review and have been withdrawn from the market, because the valuation carried out did not show safe use with respect to human health and the environment. Presently, the evaluation, marketing and utilization of whole categories of pesticides (insecticides, fungicides, herbicides, *etc.*) in plant protection among the members of the European community are regulated under Council Directive 91/414/EEC. In conclusion, IPM programs encourage the use of selective pesticides as the last control option to be applied when other management tools are not available or effective.

### 2.2. Bio-Pesticides 

The term bio-pesticide has only recently been proposed to describe those insecticides that are efficacious against the target pest but are less detrimental to natural enemies [29,30]. Traditionally, the term refers to soaps/detergents, oils and botanicals of natural origin but now includes, new systemic insecticides, insect growth regulators and products containing, micro-organisms or its components. The most important bacterial pathogen used as a biological control agent is *Bacillus thuringiensis* (Berliner), Bt., which is a gram (+) bacterium that is pathogenic to larvae. The infection is caused by the endospore of the species having crystalline inclusions consisting of one or more insecticide protein known as d-endotoxins or Cry-proteins. Because of its high selectivity, Bt is mostly used in apple IPM programs for the management of various moth species such as leafrollers [31,32,33,34]. Bt products currently available have limited effectiveness against many orchard pests due to the pest cryptic life habits [34]. The first products against the lepidopterous larvae were based upon two subspecies of *B. thuringiensis*, var: *kurstaki* and var: *aizawai* or a compounding of the two. The second generation products are grounded on the union of the two subspecies, while the third and fourth generation products are founded on new Bt. strains based on recombinant DNA technology [32].

Virus disease play important role in the natural regulation of insect population and their potential as biocontrol agents were investigated since early 1950. The most promising virus agent for controlling apple arthropod pests is a granulovirus (family Baculoviridae), which was first isolated from dead larvae found in an orchard in Mexico [35]. Granulovirus of codling moth (CpGV) is insect-specific granulovirus that offers new means of highly selective control of the codling moth *Cydia pomonella* L in fruit orchards [36]. The commercial products that are utilized in IPM programs contain the virus in an aqueous suspension and are sprayed during the egg hatch. Thus, timing of GpCV application is critical in order to promote ingestion of the occlusion bodies into neonate larvae before they are entering into the fruits. The GpGV is highly selective and host range is actually limited to *C. pomonella* and few tortricidae [26,37]. Commercial products of CpGV are now registered and available in Europe and North America and used by orchardists worldwide [37]. In addition, the application of CpGV is compatible with the use of other compounds in IPM [37,38]. Its sensitivity to degradation by UV light is the most important limitation for its widespread use as a biological control agent and research is being carried out to solve the problem [39]. The combined use of yeast-based attractants, feeding stimulants or pear ester is proposed as novel insect control technique to enhance larval mortality when using granulovirus [40].

The insecticidal properties of nematodes have been as well tested and have therefore become increasingly popular in IPM programs [41]. Two species have been commercialized, *Steinernema* and *Heterorhabditis* (Rhabditida) which are insect specific parasitic nematodes and exert lethal action to their host due to the transmission of bacteria. *Steinernema carpocapsae* (Weiser) and *Steinernema feltiae* (Filipjev) are used for the control of *C. pomonella* and especially in controlling the overwintering larvae of the moth. However, *S. carpocapsae* expresses limited host search behavior (ambusher species), while *S. feltiae* is considered as an intermediate search strategist and expresses higher search capacity. In contrast, entomopathogenic nematodes have very little potential in providing control of the woody apple aphid *Eriosoma* *lanigerum* (Hausmann), probably because of the inability of their symbiotic bacteria to grow in the aphid body and to produce the lethal endotoxins [42].

The main factor limiting the effectiveness of entomopathogenic fungi is their requirement of high humidity and moderate temperature for spore germination and development. Their use as biological control agent in apple IPM is still limited to application of *Bauveria bassiana* (Basi) to control larvae of codling moth and apple clearwing moth, *Synanthedon myopaeformis* (Borkahousen) (Lepidoptera: Sesiidae) [43].

### 2.3. Semiochemicals, Mating Disruption and Mass Trapping 

Semiochemical-mediated communication affects several types of interactions in organisms in natural habitats [44]. Likely the first and the most representative examples of semiochemicals application in IPM programs is the use of sex pheromones in detection and monitoring population emergence [45]. Captures in traps baited with synthetic pheromone lures accurately show whether a specific insect is present, and when its seasonal flight period starts. A simple and widespread strategy is to time insecticide sprays accordingly [45]. Besides, the pheromone chemical identification and the discovery of the physiological mechanisms regulating olfactory perception and behavior in insects have paved new ways to control the economically important pest species [46,47].

This know-how has been successfully used to develop direct control means whose applications outcompete conventional insecticides [48]. The primary aim is interfering with male-female sexual communication by permeating the atmosphere with synthetic pheromone and to bring down the likelihood of successful mating. Pheromones need to be formulated to prevent degradation in the field and to release them slowly into the atmosphere, providing a constant release of adequate levels of pheromone throughout the growing season while adult moths are present [46,47,48]. A wide range of pheromone delivery systems have been developed and made available for the growers (*inter alia* [49,50,51,52]). Microcapsules and hollow fibers, deployable via air-blast sprayers, were developed to fit the assumption that high density point sources were needed to uniformly permeate the atmosphere with pheromone. The main shortcoming of these formulations is the short lasting pheromone release that requires the repetition of the applications during the season. A second approach is represented by the hand-applied reservoir-type formulations, generating a plumes of high concentration of pheromone and that required to be applied at a rate of hundreds dispensers per hectare. Their season-long pheromone emission allowed us to guarantee the required amount of pheromone with a single application, but the cost of labor associated with hand application has been cited as an impediment to broader adoption of mating disruption [53].

More recently, low-density, high-release dispensing systems, emitting an aerosol spray of solvent-diluted pheromone of electronic circuit-controlled intervals, have been developed. These pheromone delivery devises are installed by hand, at a density of 2-5 units per hectare with a consistent reduction of the application labor cost compared to hand-apply hundreds of passive reservoir dispensers. Pheromone mating disruption have been shown to produce reliable results especially in area-wide programs [51,54,55] taking advantage from treatment of large contiguous blocks of orchards. In fact, potential weakness in using discrete point source of pheromone is that it may leave areas of little or no pheromone coverage, where mate finding may occur [52]. Mating disruption has been first implemented for the management of the key pest *C. pomonella* and its efficacy is widely confirmed [40,56,57,58]. However, the consequent reductions in the number of insecticide sprays needed for adequate control of the key pest have resulted sometime in outbreaks of secondary tortricid pests such as leafrollers. To tackle this problem, companies have developed multispecies disruption formulations, which are now available for growers, allowing concurrent management of co-occurring key and secondary pests [59,60,61].

Among the major advantages of pheromone mating disruption technology is that it does not exhibit adverse effects on non-target pests and it can be used to manage pest resistance. This has pushed its worldwide application; the estimated orchard area treated with codling moth mating disruption has been reported to exceed 200,000 ha of which nearly 70,000 ha are in Europe [45]. Less extended is the European orchard area treated with MD for secondary apple pests like oriental fruit moth *Grapholita molesta* (Busk), small fruit tortrix *Grapholita lobarzewskii*, (Nowicki), leafrollers *Adoxophyes orana* (Fischer von Röslerstamm), and *Pandemis heparana* (Denis and Schiffermüller)*, Spilonota ocellana* (Denis & Schiffermüller), clearwing moth *Synanthedon myopaeformis* (Borkhaousen), leopard moth *Zeuzera pyrina* (L.), even though either specific or multispecies disruption formulations targeting these secondary pests are commercially available.

Mass trapping (MT), using lures to catch the maximum number of fruit flies, has been devised many years ago [62] and it was occasionally practiced for many years though not always the best effect. However, the technique has been used in apple for *Ceratitis capitata* Wiedemann [63] and for pests such as *Cossus cossus* L. [64] and *S. myopaeformis* [65].

Over the last decade, the efficiency of mass trapping technique used against *C. capitata* (medfly) has been confirmed in several fruit crops [66,67,68,69,70,71] including apple [63,70]. The spread of the use of this technique has been possible thanks to the development in the last decade of the 20th century of dry food based attractants with the aim to use them to monitor male and female medfly populations in order to calculate population dynamics [71]. These attractants compete effectively with apple to attract flies, which once they have entered the trap are killed by an insecticide [72]. In areas where medfly is a key pest in apples, mass trapping is used as the main system for the pest control and it is integrated into IPM programs. More recently, a device of attract and kill using the same attractants than mass trapping but in an envelope impregnated with an insecticide in the outside, was also tested to protect apple production from medfly damage [73], although more studies are still necessary to set its efficacy in apple under different population levels and varieties. Anyway, in both systems, mass trapping and attract and kill, chemicals are utilized only when the population increases considerably, as reinforcement [67,74].

### 2.4. Biological Control

A wide literature documenting the effective role of natural enemies in controlling apple pests has been published and it is mainly focused in the study on conservation biological control of a wide array of arthropod pest like mites, aphids, scales, moths and leafrollers (*inter alia*, [75,76,77,78,79,80,81,82,83]). Hence, it is out of the scopes of the present work to revise these fundamental studies that constitute the conceptual cornerstone of integrated pest management. On the other hand, integrated pest management programs emphasize the more pertinent integration of biological and chemical control, to maintain pest populations below economic thresholds [11]. Combination of biological and chemical control tactics is not always compatible; insecticides can disrupt natural enemies through lethal and sub-lethal means causing pest resurgence or secondary pest outbreaks [84,85]. Compatibility between chemical and biological control depends on the chemistry and the inherent toxic properties of the pesticide in relation to the physiology of the pest and its natural enemy, on the application timing in relation to the life stage susceptibility of the natural enemy, and the actual presence of the non-target organism in the treated field or tree canopy. As combining tactics may provide better long-term sustainable pest suppression, IPM programs need to contemplate strategies able to reduce natural enemy exposure to insecticides.

Potential mitigation measures applicable at the apple farm scale include selective insecticides, low dosage, timing of application, special formulations, site-specific applications [86]. Parasitism in codling moth can be improved by better targeting of pesticide treatments [87]. Earwig populations for example are strongly affected by orchard management, suggesting that these insects could be used as a bioindicator of the intensity of the orchard management [88].

Improvement of mite biological control has been reached by replacing broad-spectrum pesticide with more selective insecticides [89,90]. Yet, the combination of a different mode of action of insecticides is a challenge in apple production which should take into account market requirements for residues, the permitted active ingredients and the need to avoid resistances. Due to these factors, the use of pyrethroids has increased the last years, mainly to control mite/insects populations close to harvest. It is well known that pyrethroids can induce outbreaks of spider mites [91] because they are harmful to phytoseids living in fruit trees [92]. This fact has challenged the natural biological control of tetranychids in apple trees in the EU countries. In the USA, New Zealand, and Canada have been reported on the effectiveness of releasing resistant strains of Phytoseiidae to control spider mites [92,93,94]. In Europe, resistant strains of *Typlodromus pyri* (Scheuten) and *Amblyseius andersoni* (Chant) to pyrethroids have been found from vineyards in the South-West of France [95,96,97]. Both species are the main predatory mites of *Panonichus ulmi* (Koch) in the European countries.

Concerning the effect of different apple management systems (organic, IPM and conventional) in beneficial mite composition, recent studies [97], related the type of management system to the Phytoseiidae composition but not to their abundance. The same study reports *A. andersoni*, *Euseius finlandicus* (Oudemans), and *T. pyri* as the three most common species, while the total phytoseiid abundance in the orchards with different pest management systems did not differ. Moreover, the relative abundance of *A. andersoni* increased with the pesticide load of the orchards whereas the relative abundance of *E. finlandicus* decreased. Nevertheless, beside the effect of different management strategies on apple mite composition [97], other factors, such as competition between species and apple variety may affect the composition of beneficial mites in apple orchards. Phytoseiidae species richness, for instance, is closely related to the presence or absence of domatia in the leaves [98]. In those cultivars where few or no phytoseids are found other strategies need to be developed to conserve the generalist predator like *Anystis baccarum* (L.) [99], a species which was reported to be the most frequent predatory mite in apples in Northern Ireland. Besides its role in controlling tetranychid pests, it has also been reported predating leafroller larvae in apple in New Zealand [100]. These new approaches to improve the biological control of mites in apples started to play a major role in the IPM design for different varieties and regions and it shows that more studies are still needed on the role of generalist predators in apple orchards.

The transition from conventional fruit production, which used broad spectrum insecticides, to integrated fruit production which was based on selective insecticides produces effects on the pests and natural enemies’ populations. In New Zealand apple orchards, for example, this transition was initially accompanied by a surge in *E. lanigerum,* and this was followed by a slow colonization of its parasitoid *Aphelinus mali* (Haldemann) which gradually have reduced the aphid population to acceptable levels [101].

The efficacy of biological control in apple orchards has also been improved by the use of new spray application techniques. Low dosage of pesticide, of alternate row-middle spraying technique, left untreated refugia for *Stethorus punctum* (Casey) and was a key factor for successful biological control of mites [102].

Furthermore, recent advances in geostatistical analysis provide means to identify hot spots of high pest intensity, direct the pesticides applications and preserve natural enemies [103]. Moreover, the use of risk assessment maps, provided by the geostatistical analysis of the pest distribution, was incorporated into an apple IPM program to allow site–specific application of pesticide and minimizing direct control tactics to the codling moth. Creation of refugia and improvement of the quality of natural habitat within the farm and at the landscape scale, help to protect biodiversity and provide ecosystem services like biological control [104,105]. The use of hedgerows surrounding apple orchards as well as planting flowering plants strips improve the landscape diversity and support beneficial insects [106,107,108] as well as the abundance and diversity of predatory phytoseiid mites [107,108]. The intercropping of some aromatic plants in apple orchards can enhance the biological control of *A. orana* and other tortricids [109]. Predator complex abundance was positively correlated with increasing alternative prey availability in a manipulated habitat of an apple orchard floor [62]. Use of compost in an apple orchard ecosystem is beneficial to management of weed, fungal, and insect pests. Populations of spotted tentiform leafminer *Phyllonorycter blancardella* (Fabricius) and migrating woolly apple aphid nymphs were reduced in the compost plots [110].

Releases of natural enemies have also been used to control mites, leaf rollers, codling moth and aphids although it is labor intensive and costly and results had not always been successful [111,112].

### 2.5. Phenology Models, Economic Thresholds and Decision Support Systems

A typical life cycle of an insect describes the phenological forms of a species with normally distributed emergence that takes place over a growing season, in which usually more than one generation is observable. Phenology models are short abstractions of the relation between temperature and population development and are used to predict the exact time of the phenological development of pest populations. Probably the simplest case of phenology model is that which is based on a non-linear regression function that correlates cumulative moth catches to heat accumulations [113]. These models are almost useful in predicting the date of the issue of the first flight or the peaks of emergence of the successive generations and are actually empirical regression models which present the best mathematical fit and do not have any clear inherent meaning. Even so, they offer useful insights into critical time windows for which specific management activities should be packed away. Hence, they are utilized for decision making and particularly for the placement of MD pheromone dispensers, timing of pesticides and especially the application of biorational compounds that express selectivity to specific developmental stages (e.g., bio-pesticides and Insect Growth Regulators).

Moreover, phenology models can be used along with Economic Injury levels [EIL]. The EIL belongs to the most basic of the decision rules; it is a theoretical value that, if actually attained by a pest population, will result in economic damage [114,115]. However, it is noteworthy to state that many factors have limited both the design of new economic thresholds as well as the development of existing ones (for a thorough review refers to [114]. The issue may be more complex considering no tolerance for damage in fruits [116].

Nevertheless, a challenge for a wider applicability and application of models and thresholds, if available, under a routine field conditions basis is to use these mathematical descriptions across locations and real environments in an automatic manner [117]. This enables the evolution of computer software programs, as described below, to run the models and facilitate the practical application by understanding population dynamics and dissemination of pest forecasts for timely pest management decisions [118]. Thus, web-based decision support systems are becoming popular and in the future may become an absolute demand for local, regional/area-wide and international implementation of IPM systems towards sustainable Agriculture.

### 2.6. Improvements in Spray Technology

Despite apple production system was one of the first implementing the IPM principles, fruit protection still requires one of the highest numbers of spraying operations per year that are generally performed with air assisted spray machine. If the final goal of IPM is minimizing the use of pesticides and their potential adverse effects on human health, wildlife, soil water, and overall environmental quality, we cannot neglect the role played by the sprayer technique in optimizing the average deposit required for acceptable biological efficacy whilst minimizing spray losses and contamination of users, bystanders and the environment. The importance of the performance of spraying equipment for the safety and efficiency of pesticide use is also recognized by the enforcement of the European Union directive on the Sustainable Use of Pesticides (EC, 2009c), that make necessary to inspect all sprayers employed for professional use in the European Union at least once by 2016 and to establish periodical mandatory inspections schemes in all EU countries [119].

Replacement of the fine or very fine hollow cone hydraulic nozzles operated at moderate to high pressures with air induction cone nozzles, which produce very coarse spray qualities, significantly decrease environmental impact by mitigating the spray drift [120,121,122]. Spray drift in the orchard strictly depends also on sprayer air setting, both in terms of velocity and direction, so a proper adjustment of air parameters is useful not only to increase treatment efficacy, but also to prevent risks of environmental contamination. In addition to adjusting the sprayer by setting it in advance to suit the particular orchard, spray equipment should be fitted with sensors so that the adjustments are done automatically in real time during spraying. Numbers of canopy sensing systems (photocells, ultrasonic sensors, spectral sensors, laser technology) have been developed in order to instruct the sprayer to avoid spraying in absence of the target or cutting down on spray volume where the crop canopy is thin or the foliage is very poor. Implementation of these innovative technologies has generally provided a considerable contraction of chemical input and reduced spray loss due to drift and run-off [123,124,125,126,127,128]. For a comprehensive review on innovative spraying technology in Europe and the future directions of the research on this topic see also [129].

### 2.7 Innovation in the Tree Training 

Over the last 60 years, large apple trees once constituting the extensive orchards, have been replaced by dwarf trees, ideally not higher than 4 meters and planted in rows 3.5 to 4 meters apart. In these modern apple orchards, applications of chemical control are still widely based on axial fan airblast sprayers which produce a large radial spray plume resulting in high spray losses to the ground and as spray drift. A new direction in tree training is what is called “pedestrian fruit wall” that is similarly to modern vine grape plantings. This new multi axis tree training system [130,131], with more and shorter, branches than the spindle, forms close “fruit walls”. The pedestrian fruit wall training system, with its narrow canopy (1 meter wide) and its reduced height (about 2.5 meters), opens entirely new possibilities in terms of mechanization of cultural practices (pruning, thinning, weeding, harvesting), thus reducing the input of growth regulators and facilitates the introduction of innovative spraying technology like different design of tunnel sprayers [132] or the Solid Set Canopy Delivery System [133]. Both these approaches are really effective in reducing spray losses, pesticide drift and number of fungicide treatments by optimizing timing applications and improving foliar coverage. Moreover this pioneering training system permits the application of the single-row multi-functional nets, named Alt’Carpò [134,135] that has been already proved to be effective in controlling codling moth, but that can be also used to replace chemical thinning when early applied during the blossom period.

## 3. Integrated Fruit Production and Technical Guidelines

**Figure 1:**
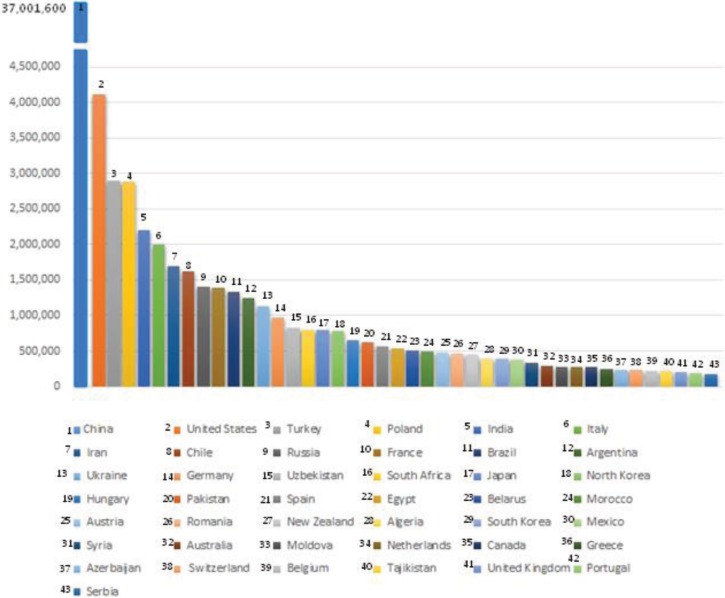
Worldwide leading countries in apple production. Rank is given per ‘legend line’ and stated as average tons for 2012 (source: FAO).

**Figure 2:**
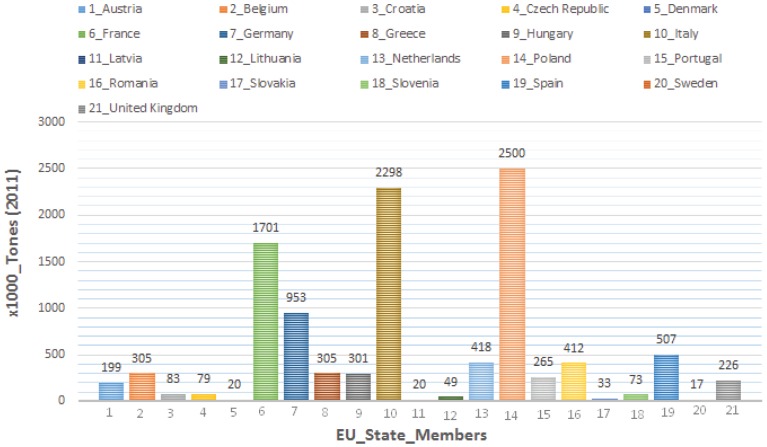
Apple production by country among the 27 EU state members 2011 (source: FAOSTAT, WAPA).

Apple production is considered among the most dynamic crop production systems. Figure 1 illustrates the worldwide average regional apple production (China and US are leading countries worldwide) [136]. The total apple production in Europe reached almost more than 12 million tons (Figure 2). In most cases, such considerable production entails a very high use of pesticides. Fruit trees, actually, are the second crop in terms of pesticides quantity used by hectare and are only exceeded by vineyards [137]. This high volume of pesticides application causes frequent presence of residues in apples and has several environmental implications, such as groundwater pollution and cases of human and animal poisoning. Concerns about these consequences have increased interest in the development IFP and application guidelines which incorporate IPM and general actions that have little or no impact on humans, beneficial organisms and sensitive ecosystems [138,139].

To date, the International Organization for Biological Control has provided the leadership for developing the philosophy Integrated Production, the general guidelines, and crop specific technical guidelines [140]. Agreeing to the IOBC definition Integrated Apple Production is a farming system that produces high quality fruits by using natural resources and regulating mechanisms to replace polluting inputs and to secure sustainable farming. “Emphasis is placed on a holistic systems approach involving the entire farm as the basic unit, on the central role of agro-ecosystems, on balanced nutrient cycles. The preservation and improvement of soil fertility and of a diversified environment are essential components. Biological, technical and chemical methods are balanced carefully taking into account the protection of the environment, profitability and social requirements”.

IOBC Guideline for IFP provided a rigorous foundation for development of sustainable utilization of pesticides legislation in the EU. Integrated Fruit Production in pome fruits has been regularly utilized to launch IFP programs in a number of European rural areas and along with pesticides properties databases can be used for environmental sound decision making [139,140]. The IOBC sets out general principles, minimum measures and guidelines for IFP in the geographic regions tracked by the IOBC/WPRS. These Guidelines are meant as a framework for the preparation of specific regional or national guidelines and measures according to IOBC standards and to facilitate their harmonization [139,140].

In general the IFP guidelines contain the following sections: definitions; professional trained and environmentally and safety conscious growers; conserving the orchard environment; site, rootstocks, cultivar and planting system for new orchards; soil management and tree nutrition; alleyways and weed-free strip; irrigation; tree training and management; fruit management; integrated plant protection; efficient and safe spray application methods; harvesting, storage and fruit quality; post-harvest chemical treatments; mode of application, controls, certification and labeling [139,140]. Nevertheless, the guidelines may be countrywide, but more often are tailored to the specific conditions of a developing area. Switzerland probably belongs among the most rigorous IFP example and about 85% of the Swiss apple farms certified as practicing integrated fruit production (IFP). Moreover, as an outcome of the latest European directives the market demand on IFP is steadily increasing and during the last years, almost 50% of the apple and the pear acreage in Western Europe are managed under an IFP program [16]. Since 1991, the cultivated apple area has increased over 40%, while the adoption of IFP in larger areas has led also the means for marketing IFP labeled fruit and which resulted up to 30% decrease in conventional pesticide application and increased usage of biorational and environmentally friendly pesticides.

However, a part of the mentioned official IFP definitions and guidelines, the regulations used by each country or organization vary and they are not always consistent with the IOBC Guidelines. The overall IPM standards, unlike organic production, are not precisely defined or legally specified in an EU or international agreement and the level of guidelines implementation varies considerably among the state members [137]. For example there are no common requirements which are imposed on growers. Instead there are many certification and market authorities which set up specific standards create IPM labels offering economic advantages to corporations and individual growers [137].

## 4. Present and Future Threats to IPM

### 4.1. Pesticide Resistance

Among the possible threats that jeopardize the effective implementation of IPM in the European apple production system, the risks related to the development of pesticide resistance and the introduction of new invasive pests deserve to be discussed.

The development of resistance to pesticides by apple pests as a consequence of increasing frequencies of chemical treatments is known since long time [141]. Pesticide resistance management is an effort to slow or prevent the development of resistance; it relies on pest management and pesticide use strategies to prolong the effective life of pesticides and it is a tactical part of the IPM. 

The codling moth, *C. pomonella*, is a worldwide pest in apple orchards. For many years, pesticide applications have been the dominant tools used for its control. However, increasing frequencies of chemical treatments led to the acquisition of resistance to many of the recommended pesticides belonging to various chemical groups. In this species, the first case of resistance was the resistance to arsenates reported by Hough in 1928 in the USA. Since then, new cases of resistance have been and are being reported in almost all of the main apple-growing regions worldwide. In Europe, insecticide resistance in the codling moth was firstly detected in the 1990s with the emergence of resistance to diflubenzuron in Italy and southeastern French populations [142,143,144]. Failures of control with pesticides were further observed in Switzerland and Spain [145,146]. Currently, the resistance spectrum of some of these populations has dramatically increased to include avermectins, benzoylureas, benzoylhydrazines, neonicotinoids, organophosphates, macrocyclic lactones, pyrethroids [146,147,148,149,150,151] and also biopesticide, like granulose virus [152].

Unlike the codling moth, in the European apple orchards the cases of insecticide resistance within the leafroller species are only sporadic. A reduction of susceptibility of the summer fruit tortrix moth, *A. orana*, to chlorpyrifos was first detected in England comparing a population regularly treated with the organophosphates and one collected in an orchard that never received the insecticide [153]. A larger spectrum of insecticide resistance in the same species was detected in two field populations in Switserland; this resistance concerned the benzoylureas hexaflumuron and lufenuron, the benzhydrazides tebufenozide and methoxyfenozide, as well as fenoxycarb [150,154,155] while these populations were only slightly resistant to chlorpyrifos-methyl [156]. No other cases of resistance are reported neither for A*. orana* nor for other leafroller species infesting the European apple orchards.

The implementation of integrated resistance management strategies is of the utmost importance to avoid further spread of the phenomenon. Moreover, rotational use of non-cross-resistant insecticides would benefit fitness costs associated with resistance [157]. This includes the use of semiochemicals and biocontrol agents. Evidence of the efficiency of mating disruption, both in term of pest control and reduction in selection pressure, is already reported in the European apple orchards [158]. Where conditions for effective application of mating disruption are not optimal, strategies such as treatments with CM granulosis virus have already demonstrated their efficiency to reduce the selection pressure of chemical insecticides [159].

Apart from insects, spider mites have historically demonstrated a propensity for developing resistance particularly in tree and vine crops. *Panonychus ulmi*, the European red mite (ERM), is the main Tetranychidae member that causes major damage in apples and, together with *Tetranychus urticae* (Koch)*,* it has been reported among the top 10 resistant arthropods [159]. Resistance in ERM was reported for the first time by Newcomer and Dean in 1952 [160] to parathion and since then, many other compounds had been included in the list: Dicofol, tetradifon, propargite, cyhexatin, clofentezine, hexythiazox, pyridaben and fenpyroximate, fenbutatin oxide, tebufenpyrad and fenazaquin, parathion-methyl and bifenthrin. Nowadays, many of these compounds have been banned by EU normative, and other active ingredients are increasingly being used to control ERM populations. Resistance to chlorpyrifos and lambda-cyhalothrin has been reported in Turkey [161] and to spirodiclofen in south-western German populations [162] and in one spot in Belgium; they have also found resistance to clofentezine in Poland populations.

Other mites can also cause damage in apple, e.g., *T. urticae* and the apple rust mite *Aculus schlechtendali* (Nalepa). *Tetranychus urticae* is the most studied mite regarding its resistance to agrochemicals, mainly because it is highly polyphagous and can damage a wide variety of crops. The apple rust mite can also cause damage in apples arriving to affect the yield [163]. Up to now, only one old report on resistance to parathion has been released in Canada for the species [164].

Concerning the management of mites in apple orchards, it is clear that the few compounds that remain effective are consequence of strict resistance management strategies [165,166] and where chemical treatments have to be combined with other measures, e.g., biological control to keep mites level under economic damage threshold. Whalon *et al*., 2008 [159] and IRAC, 2014 [167] offer a complete literature on arthropod resistance and the mechanisms involved, including the species *P. ulmi*, *T. urticae* and *A. schlechtendali* and this information has to be used to properly design the management of mites in an apple IPM program.

In the case of plant diseases resistance development may be more harsh and difficult to be managed. Because most IPM strategies for plant diseases have been adapted from insect biology models, with little considerations in biological differences, resistance management may not be as effective as in the case of arthropods. For instance, most fungal plant pathogens are haploid and with faster reproductive rates, compared with mostly diploid arthropods of economic significance, which affects inheritance of pesticide resistance traits. Beckerman *et al.* [168] argued that the actual approaches used to implement IPM, using powerful new fungicides, applied often at lower doses, have contributed to fungicide resistance problems and may still be driving that process in apple scab management.

Scab, caused by *Venturia inaequalis* (Cooke) winter, is the main disease of apple and requires numerous fungicide applications, especially where frequent rains and fairly high temperatures occur during the first growth stages. As a result, the impact of fungicide resistance in apple scab has been significant, with fungicide resistance having been identified in all classes of curative fungicides used for the control of scab. The reiterated use of fungicides to control this apple key disease threats their effectiveness and endurance over the time. Among the most recent chemical families of fungicides, activity reduction has been observed for the anilinopyrimidines (AP), the demethylation inhibitors and respiration inhibitors at the Qo site (QoI) [169,170,171,172].

To minimize potential treatment failures due to resistance events, reliable control of apple scab epidemics can be achieved by the integrated application of all types of countermeasures including the use of scab resistant varieties, biological control agents, and appropriate agronomic and hygienic practices. Contrary to arthropods pesticide resistance management, non-chemical methods of disease control are often weak or not available, so the application of fungicides of different modes of action together with the use of forecasting models, for correctly timing of treatments, is the predominant strategy in order to overcome resistance problems.

### 4.2. New Pest Threats

In recent years, the international trade in fruits and vegetables has been pointed out for a substantial growth in the volume. Europe together with the United States, and Japan are the largest importers of fruits and vegetables. Despite the international cooperation and regulatory systems put in place to inhibit the spread of plant pests, the international trade of fruits and plants is the main responsible for facilitating their spread.

Among the potentially invasive species endangering European apple orchards, Brown Marmorated Sting Bug (BMSB) *Halyomorpha* *halys* (Stål) (Heteroptera: Pentatomidae), is probably the one that deserves the highest attention [173]. *Halyomorpha* *halys*, is an herbivorous insect species that was accidentally introduced to the United States with international commerce, *i.e.*, via bulk freight containers from either Japan, Korea, or China [174] where it has caused serious economic injury to many crops, including tree fruit [175,176]. Losses caused by *H. halys* fruit injuries in apple orchards in the mid-Atlantic states have increased dramatically in recent years and many fruit growers have implemented an aggressive management programs to control this pest resulted in up to an approximately fourfold increase in insecticide use [175,177]. Insecticide array included broad-spectrum insecticides in the postbloom period has also increased the incidence of secondary pest outbreaks [174].

Additional invasions have been detected also in Europe, namely in Switzerland, Liechtenstein, Germany, Italy, France, Hungary and Greece [176] suggesting this invasive species could emerge as a cosmopolitan pest species. *Halyomorpha halys* has been included in the EPPO Alert List for more than 3 years [177]. In 2013, it was considered that sufficient alert has been given and the pest was deleted from the Alert List. (EPPO). Even though apple injuries caused by *H. halys* has not been reported in Europe so far, damage to apples may be under-reported because it can be confused with cork spot or bitter pit, two physiological disorders caused by calcium deficiencies [174]. Based on the US experience [175,177], chemical control with broad spectrum insecticides is expected to be the essential component of the management reaction against this newly invasive species once established in the apple orchard. These aggressive management programs could have detrimental ecological consequences that could jeopardize the successfully implementation of IPM.

## 5. What Has Been Changed in Market Requirements and Residues?

The total apple production in Europe is estimated to be close to 12 million tons in 2014 [178]. While production is confirming the constant increase reported in the recent years, the consumption *per* *capita* is decreasing at the rate of 2% per year and it accounts now for 15 kg/person per year. The apple production system is also challenged by a loss of competitiveness due to high labor costs in relation to other regions of the world and the political goal to significantly reduce pesticides use. In addition, consumers and the markets that supply them are demanding a large reduction, ideally the elimination, of pesticide residues on fruit.

In fact, the consumer's attention to the potential risks to health and the environment arising from the use of pesticides in agriculture is growing and despite checks by regulatory authorities attesting to the contrary, many consumers are instead concerned about the safety of fruit which are perceived as “residue carriers”. Supermarket chains have interpreted this concern by imposing limits on pesticide residues on fruit well below those required by the law, introducing a new element of discrimination in the acquisition of the product [179]. These constraints itself do not have a scientific justification, but purely commercial nature, however, they have led producers to speed up the review of pest control strategies in the search of technical tools that would enable to meet the requirements of the market without jeopardizing the principles of IPM, including for instance the combination of mating disruption and biopecticides [180].

Although Integrated Production does not mean a reduction in number of pesticide residues, alternative systems to pesticide use are recommended and expected by both EU regulation and markets, to improve the quality of agricultural production and environmental safety and to meet consumers’ expectations for safety apples.

As an example of how the apple production system has included the pesticide residue issue in its IFP strategies, we report here the cases of the Trentino (Italy) and Girona (Spain) regions. In Trentino apple production covers 10.500 ha and produces 500.000 tons/year representing nearly 5% of the European apple production. Guidelines for integrated fruit production are implemented since 1991 [181] with the support of a technical advisory service that is available for the growers free of charges. A technical committee oversees the updating of the guidelines and takes into consideration the results of the survey conducted annually by the growers association to detect the level of 406 pesticide residues. Fruit sampling at harvest is annually carried out in order to analyze both the number of active ingredients (a.i.) and level of residues present on the fruits. In the last three years, the average number of a.i. found in the sample collection ranged from 3.18 (2011) and 4.55 (2010). In general, given the climatic characteristics of the region, pesticide residues on fruit are mainly represented by fungicides used for the apple scab control and for the prevention of storage diseases. Table 1 shows the number of samples analyzed annually over the last seven years, the percentage of samples with residues of insecticides/acaricides, as well as the distribution of the samples in four classes according to the percentage of residual insecticides/acaricides that has been observed compared to the maximum residue limits (MRLs).

In recent years, there has been a steady reduction of insecticide/acaricide residues in so much that in 2011 two thirds of the samples were free of insecticide/acaricide residues, and if the residue was present, 99 times out of 100 it was below 30% of the MRL. This positive results are a consequence of a careful management of pests, based on field scouting and visual controls performed by the consultants, application of tolerance thresholds when available and extensive use of mating disruption for the control of the codling moth *Cydia pomonella*, the main pest in this area.

**Table 1:** Percentage of samples with insecticide/acaricide residues and percentage distribution of samples with respect of pesticide maximum residue levels (n: number of samples analyzed over seven years)

	Year						
	2005	2006	2007	2008	2009	2010	2011
	*N*						
**Residue analysis**	*560*	*725*	*584*	*603*	*607*	*617*	*575*
**% of sample with residue up to 30% of MRL**	96.3	95.4	96.6	95.2	97.2	99.6	99.0
**% of sample with residue between 30% - 50% of MRL**	3.0	2.9	1.5	2.7	2.0	0.2	0.0
**% of sample with residue between 50 and 100% of MRL**	0.7	1.4	1.2	1.2	0.6	0.0	0.5
**% of sample with residue more than 100% of MRL**	0.0	0.3	0.7	0.9	0.2	0.2	0.5

**Figure 3:**
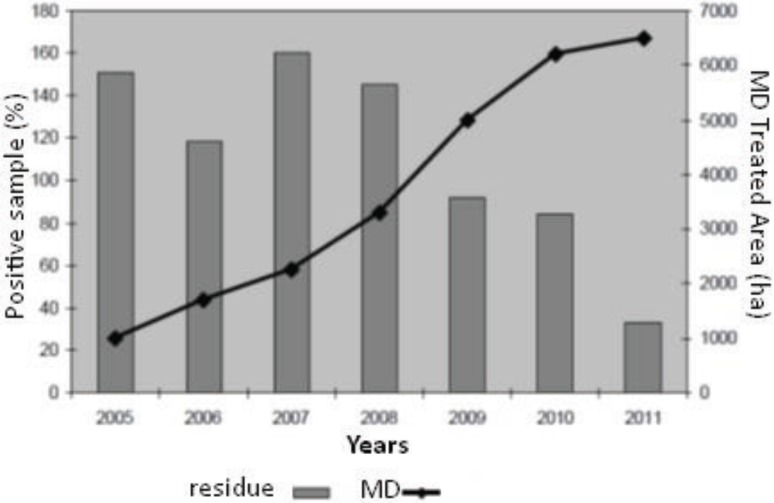
Percentage samples with insecticide/acaricide (gray bars) and area treated (ha) with mating disruption (black line) in Trentino.

Figure 3 shows the positive influence of mating disruption on the reduction of samples with insecticide/acaricide residues; the percentage of samples with insecticide/acaricide residues decreases by increasing the MD treated area. Based on these data, it was thought useful to evaluate how feasible could be producing apple without any insecticide/acaricide residues. This goal seems to be achievable in many situations, by extending the combination of the MD with biopesticide such as GpGV [180] and/or with low residual products such as emamectin-benzoate for the control of codling moth at the end of the season [182] and by promoting the use of sulphur-based-preparations for the combined control of apple scab and rust mites (RM) *A. schlechtendali* [183].

Similarly, in the Girona region, IPM started in the early ’90 and in the Catalonia region there is also an IPM committee formed by researchers, technicians and officers that update the guidelines. This committee is depended on the Government and from the beginning of IPM in the area and progressively, all the available new technologies for pests control has been incorporated at experimental orchards level until a complete program to produce apples with low residues was achieved. When results were strong and reliable regarding both pest control and residues at harvest, it was decided (2009) to implement the program in a wider area, after an agreement with the majority of the growers. In Table 2 are summarized the main arthropods and diseases and the recommended control method for each.

These control methods were chosen in function of its efficacy and also taking into account the environmental protection. The main sprays with chemicals have been concentrated when the fruits are not still present or when they are very small. In case of an outbreak close to harvest, it is recommended to use a chemical that can have an easy and quick degradation in the environment respecting the rules of resistance management. Although this program maximizes all the available tools, there still are some bottle necks; one of them is related with diseases which control are based on fungicides which in some cases can be applied as directed by the predictive models; but, in any case, these sprayings are still highly dependent on weather conditions of each year, demanding more or less sprayings. As a consequence, sometimes it is quite difficult to avoid some fungicide residues at harvest. The second bottle neck regards the new arthropods or diseases that are lately appearing. Some of them are invasive, while some others were already present in the environment and by unknown reasons started to increase their populations and damage. Nevertheless, the results of the program were highly satisfactory because it has permitted to decrease the use of insecticides up to 36.9 % and 24.5% the use of fungicides. Regarding residues level, in the 58% of the orchards in which this system was applied there were no residues and in the 42% remaining the level of residues detected was minor than the 20% of the LMR stated by the EU normative, which is very low.

**Table 2:** Common pests and diseases of apple in chronological order of appearance until harvest; right moment to start control and control methods for each.

Pest /Desease	Plant, Pest or Disease Phenological Stage	Control Method
Apple scab	From 07 (BBCH* scale)	Models to decide the right time to spray fungicides
San José scale	From 07 (BBCH scale)	Paraffinic oils and insecticides in pre-bloom.
Powdery mildew	From 10 (BBCH scale)	Fungicides
Fire blight	Pre-bloom Bloom	Bactericides based on copper in pre-bloom and microbial control during bloom
Aphids (Rosy apple aphid, Green aphid)	Pre- and/or postbloom treatments	Biological control + Insecticides
Woody Apple aphid	Pre- and/or postbloom	Biological control + Insecticides
Leafrollers	Pre- and/or postbloom treatments	Insecticides based on visual inspections (pre-bloom) and on captures in monitoring traps (post-bloom)
Codling moth	64 (BBCH scale)	Mating disruption (as a base system) plus CpGv or common insecticides based on captures in monitoring traps
Leopard moth	64 (BBCH scale)	Mating disruption (as a base system)
Mediterranean Fruit Fly	At the beginning of the flight	Mass trapping or attract and kill
European Red Mite	Pre- and/or postbloom treatments	Biological control. Miticides, only in case of not Phytoseiidae
Oriental Fruit Moth		Mating disruption (as a base system) plus insecticides based on captures in monitoring traps
Storage diseases	No fungicides	

*BBCH-scale is a host specific climax used to identify the phenological developmental stages of a plant.

## 6. Conclusions

From the initial development and leading implementation of the IPM in years 1950s, many other expectations were placed on this pest control approach beside the original aim of the reduction of the deleterious effects caused by broad-spectrum insecticides used in agriculture. Integrated Pest Management, is a decision-based operation, required the coordinated function of multiple tactics for optimizing the control of whole classes of pests (insects, pathogens, vertebrates and weeds) in an ecologically and economically sound manner. With the development of the concept of the holistic system approach where the orchard is playing the central role of agro-ecosystem, IPM became an indispensable element of the Integrated Fruit Production. The pest control strategies are more and more integrated with the use of natural resources where natural mechanisms are expected to replace potentially polluting inputs. Chemical treatments, accurately selected taking into account the protection of the worker and the consumer’s health as well as the safeguard of the environment, are still essential to control pest and weed but they are integrated with agronomic preventive measures and biological and physical methods.

This evolution of the IFP is also referred as sustainable agriculture and involves economic, environmental and social aspects. Beside the traditional economic criteria of the market-oriented agriculture, sustainable agriculture includes the assessment of the environmental impact of agronomic practices like the carbon footprint, life cycle assessment, energy use, biodiversity. Economic and environmental sustainability are strictly interdependent, as it is the entire agricultural production process with societal context where it is held. These environmental impacts generated by the fruit production are raising consumer concerns over product standards which are now not only limited at the quality and safety standards, but they are frequently including process aspects of the production system. As a consequence, consumers and supermarket chains are requiring certification of good farm practices (e.g., Global G.A.P; TESCO NURTURE).

Europe is well placed to take advantage of sustainability issues due to its long tradition in IPM and its demonstrated ability to evolve this old concept in an innovative approach required by modern society.
